# High dose of tigecycline for extremely resistant Gram-negative pneumonia: yes, we can

**DOI:** 10.1186/cc13942

**Published:** 2014-06-24

**Authors:** José Garnacho-Montero, Carmen Ferrándiz-Millón

**Affiliations:** 1 Unidad Clínica de Cuidados Críticos y Urgencias, Hospital Universitario Virgen del Rocío, Avd Manuel Siurot s/n. 41013, Sevilla, Spain; 2 Instituto de Biomedicina de Sevilla, Hospital Universitario Virgen del Rocío/CSIC/Universidad de Sevilla, Avd Manuel Siurot s/n. 41013, Sevilla, Spain; 3 Red Española de Investigación en Patología Infecciosa, Hospital Universitario Virgen del Rocío, Avd Manuel Siurot s/n. 41013, Sevilla, Spain

## Abstract

Few antimicrobials are currently active to treat infections caused by extremely resistant Gram-negative bacilli (ERGNB), which represent a serious global public health concern. Tigecycline, which covers the majority of these ERGNB (with the exception of *Pseudomonas aeruginosa*), is not currently approved for hospital-acquired pneumonia, and several meta-analyses have suggested an increased risk of death in patients receiving this antibiotic. Other studies suggest that the use of high-dose tigecycline may represent an alternative in daily practice. De Pascale and colleagues report that the clinical cure rate in patients with ventilator-associated pneumonia is significantly higher with a high dose of tigecycline than with the conventional dose, although mortality was unaffected. This high dose is safe; no patients required discontinuation or dose reduction.

## 

In this issue of *Critical Care*, De Pascale and colleagues [1] report a retrospective study evaluating a high dose (HD) of tigecycline (200 mg followed by 100 mg every 12 hours) in severe infections compared with the standard dosing. In patients with ventilator-associated pneumonia (VAP), the HD of tigecycline was an independent predictor of clinical cure. Carbapenem-resistant *Acinetobacter baumannii* and *Klebsiella pneumoniae* are the most frequently isolated bacteria [1].

Nowadays extremely resistant Gram-negative bacilli (ERGNB) constitute a real threat to humans. Nevertheless, the discovery and development of new antibiotics have dramatically fallen in the last two decades [2]. Several of these new molecules did not achieve the pre-established efficacy endpoints in clinical trials carried out in seriously ill patients [3-5]. It is likely that, at the recommended doses, these antibiotics do not achieve the desired pharmacodynamic targets when pharmacokinetic parameters are altered as occurs in critically ill patients [6]. Off-label use at higher doses may overcome these limitations and represents an alternative in real life.

Tigecycline, active *in vitro* against a wide range of Gram-negative bacilli, is approved for the treatment of complicated skin structure infections and intra-abdominal infections. Nevertheless, it is not approved for hospital-acquired pneumonia (HAP), including VAP. A randomized controlled trial concluded that, in the clinically evaluable population, the cure rates in patients with VAP treated with the conventional doses were lower than those in patients treated with imipenem. In three mechanically ventilated patients treated with conventional doses, concentrations in the epithelial lining fluid were absolutely insufficient to eliminate the culprit pathogens [7].

Recent meta-analyses have suggested increased risk of death in patients receiving tigecycline compared with other antibiotics, particularly in patients with VAP [8,9]. Nevertheless, a double-blind randomized study of patients with HAP/VAP compared two doses of tigecycline with imipenem. Numerically higher efficacy values were observed with the tigecycline 100 mg twice-daily dose relative to lower doses of tigecycline and imipenem/cilastatin in the treatment of HAP [10].

Very little information exists about the efficacy and safety of HD tigecycline. Two studies have reported its use in combination with other antimicrobials in infections caused by carbapenemase-producing *K. pneumoniae*[11,12]. The use of HD tigecycline did not influence the outcome in a small series of critically ill patients with infections due to carbapenemase-producing *K. pneumoniae*[13]. In the study by De Pascale and colleagues [1], as in previous studies [10-13], this HD was not associated with undesirable effects.

Its retrospective design, the small number of patients included, and the evaluation of a subjective endpoint (clinical cure) are obvious limitations of this study. The small number of patients in whom microbiological cure was evaluated precludes sufficient correlation with clinical results. Furthermore, various co-administered antibiotics impede a clear view of the contribution of tigecycline in the outcomes.

Valid alternatives against ERGNB are urgently needed. The only real solution is to test old or new antimicrobials in well-designed clinical trials [14]. We make an urgent plea: the design of these trials should take into account that schedule regimens of antibiotics based on data from healthy volunteers or non-critically ill patients are clearly insufficient to treat severe infections. In addition, randomly assigned patients should be representative of those who will need these drugs [15].

However, clinical research is expensive and the results take several years to become available. In the meantime, observational studies like the one by De Pascale and colleagues are of value for clinicians in the difficult challenge of choosing the most suitable therapy for infections caused by ERGNB.

### Abbreviations

ERGNB: Extremely resistant Gram-negative bacilli; HAP: Hospital-acquired pneumonia; HD: High dose; VAP: Ventilator-associated pneumonia.

### Competing interests

The authors declare that they have no competing interests.
